# Diffuse cavernous hemangioma of the skull misdiagnosed as skull metastasis in breast cancer patient: one case report and literature review

**DOI:** 10.1186/s12885-019-5341-x

**Published:** 2019-02-25

**Authors:** Huizhi Liu, Xiaojing Chang, Hua Shang, Feng Li, Huandi Zhou, Xiaoying Xue

**Affiliations:** 1 Department of Radiotherapy, the Second Hospital of Hebei Medical University, Shijiazhuang, 050000 China; 2 Department of medical imaging, the Second Hospital of Hebei Medical University, Shijiazhuang, China

**Keywords:** Cavernous hemangioma of skull, Metastasis, Misdiagnosis, Breast cancer

## Abstract

**Background:**

Primary intraosseous cavernous hemangiomas (PICHs) of the skull are extremely rare. To date, diffuse cranial hemangioma of skull has not been reported. In cancer patients, it is often misdiagnosed as metastasis.

**Case presentation:**

Here, we presented a case of a 50-year-old female patient suffering from slightly headache who received breast cancer modified radical mastectomy in 2004, computed tomography and magnetic resonance imaging findings revealed abnormal lesions of diffuse skull which were misdiagnosed as skull metastasis, and the relevant literatures were also reviewed.

**Conclusions:**

Diffuse cavernous hemangioma of the skull is exceedingly rare, and imaging data are not typical. The condition is often misdiagnosed, and pathological evaluation is necessary and important. In cases where the mass cannot be completely removed by surgery, radiotherapy could be beneficial.

## Background

Primary intraosseous cavernous hemangiomas (PICHs) are benign vascular tumors that are characterized by osteolytic bone destruction and often occur in the vertebral column but are extremely rare in the skull [1]. To date, there have been fewer than 100 reported cases of skull PICHs, and the reports were mainly concentrated in Europe, North America, and East Asia [2]. Diffuse cranial hemangioma has not yet been reported. In cancer patients, this condition is often misdiagnosed as metastasis. Here, we reported a case of hemangioma of the skull in a breast cancer patient, which was misdiagnosed as skull metastasis and a review of the literature.

## Case presentation

The patient was a 50-year-old female who underwent surgery in 2004 for mucinous breast carcinoma of the right breast [T2N1M0 (IIb)], and received postoperative CMF (cyclophosphamide+methotrexate+fluorouracil) chemotherapy, radiotherapy with 50Gy and endocrinotherapy with tamoxifen for 5 years. Unfortunately, she did not receive any follow-up examination after completion of the treatment. In 2015, she found that the right local surface of the scalp had become irregular and was increasing in size without pain or numbness. More than 1 year later, the patient experienced a slight headache which was relieved with antipyretic analgesics. Then edema of the frontal scalp and bilateral upper eyelid followed, particularly on the right side. She denied any past trauma history. The physical examination revealed the following: edema of the bilateral frontal scalp and upper eyelid, the right frontal, temporal, and dorsal scalp were slightly lumpy with normal scalp color, and the lumps were immobile and solid but without tenderness. The right thoracic wall was modified due to the prior radical mastectomy for the treatment of breast cancer and the right upper limb was free of edema. Neurological examination revealed no abnormalities other than the slight headache. No abnormalies were found in the remaining examinations. Laboratory examination: The results of routine blood and urine examinations were normal, as were those of the biochemistry examination.

**Fig. 1:**
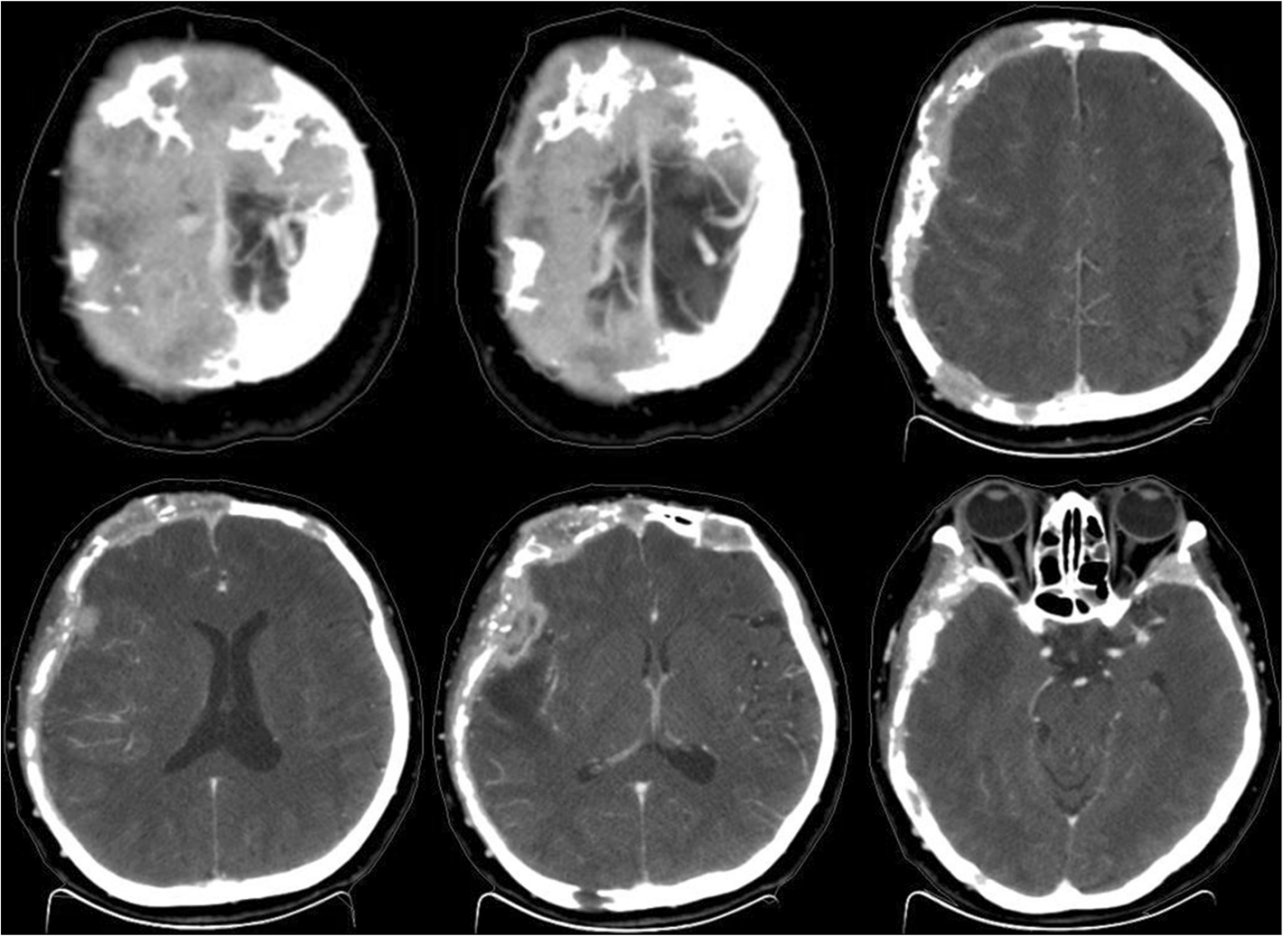
Computed tomography revealed that the right temporal lobe, and bilateral frontal lobes contained lesions. Meanwhile, the bilateral forehead, and right temporal occipital exhibited diffuse osteolytic bone destruction. The tesions had a “bean curd” type of morphology. The border of the lesion was undefined. The surrounding bone without hardening

**Fig. 2:**
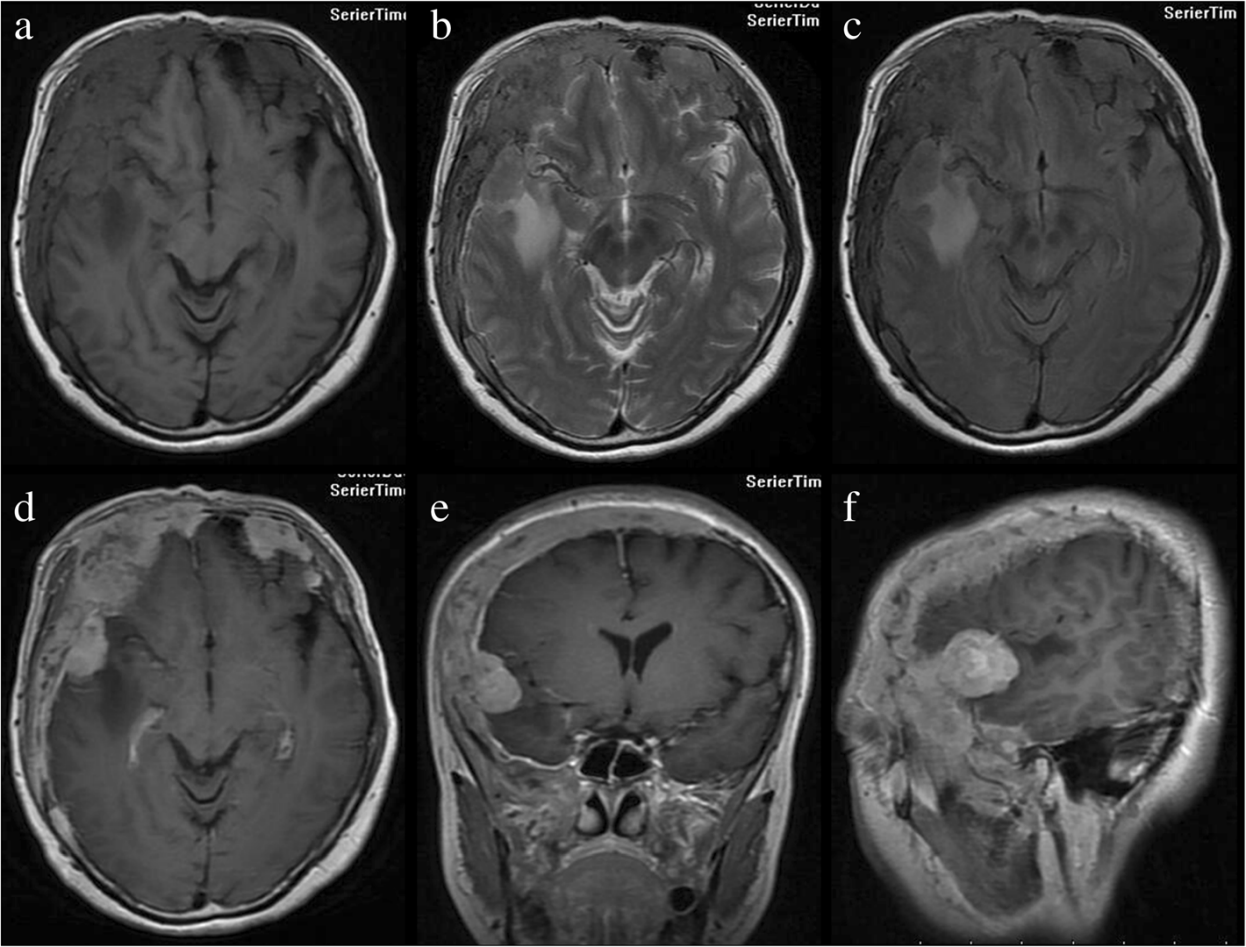
Magnetic resonance imaging revealed that thickening of the bilateral forehead, right superior temporal occipital bone and bilateral upper orbital bone. There were multiple visible flaps and nodules along with slightly longer T1 (**a**), slightly longer T2 (**b**), and other FLAIR (**c**) signals in these areas of bone thickening. An enhanced scan revealed slightly irregular enhancement (**d**, **e**, **f**). Significantly enhanced nodules could be observed on the right side of the temporal and temporal occipital meninges. The largest nodule was at the right side of the temporal lobe, and pressure spread to the adjacent brain parenchyma, around pressure exist the long T1 long T2 edema signal. There was slight compression of the adjacent brain parenchyma and ventricle

**Fig. 3:**
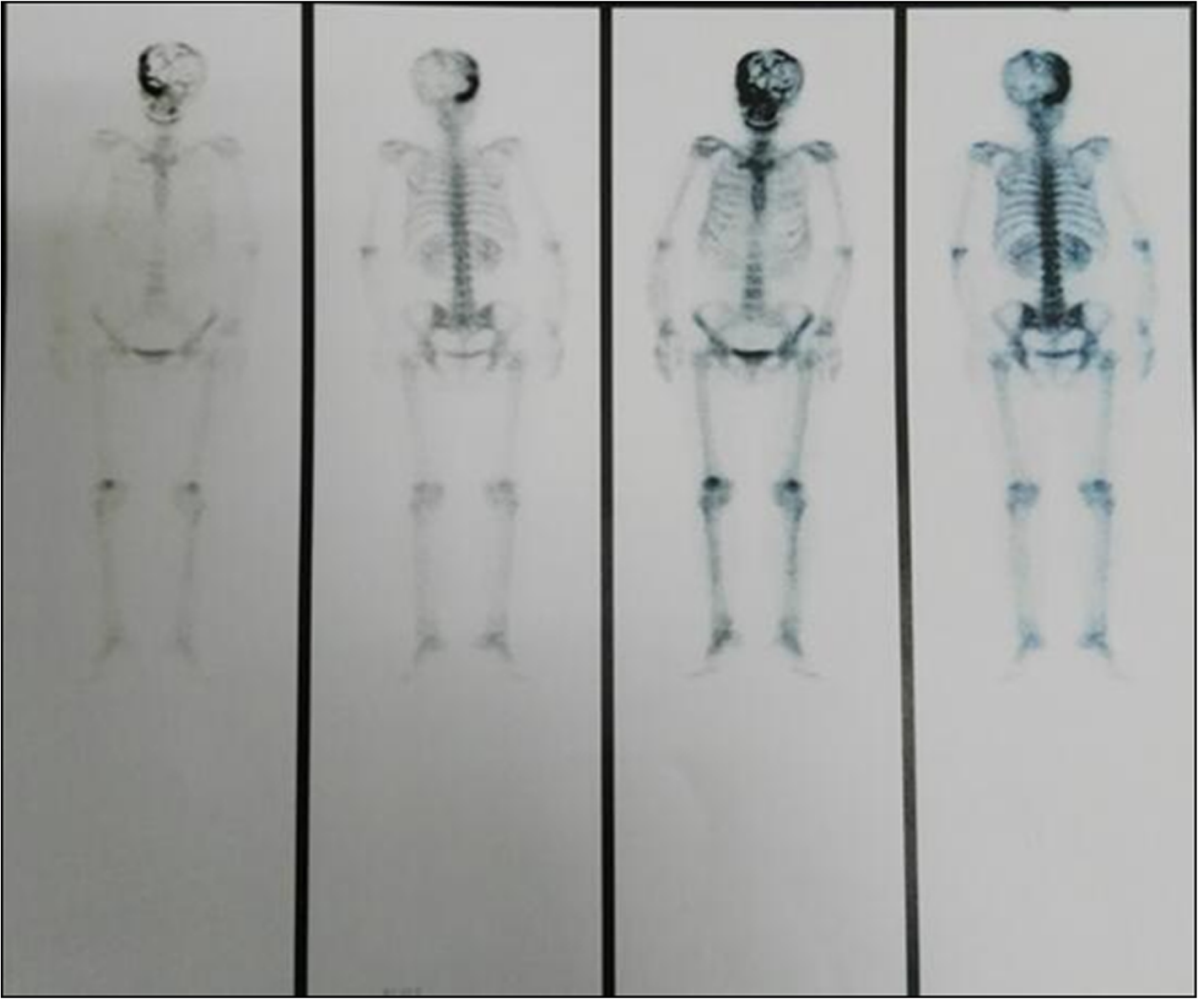
Systemic bone imaging revealed abnormal bone metabolism in the skull bone

**Fig. 4:**
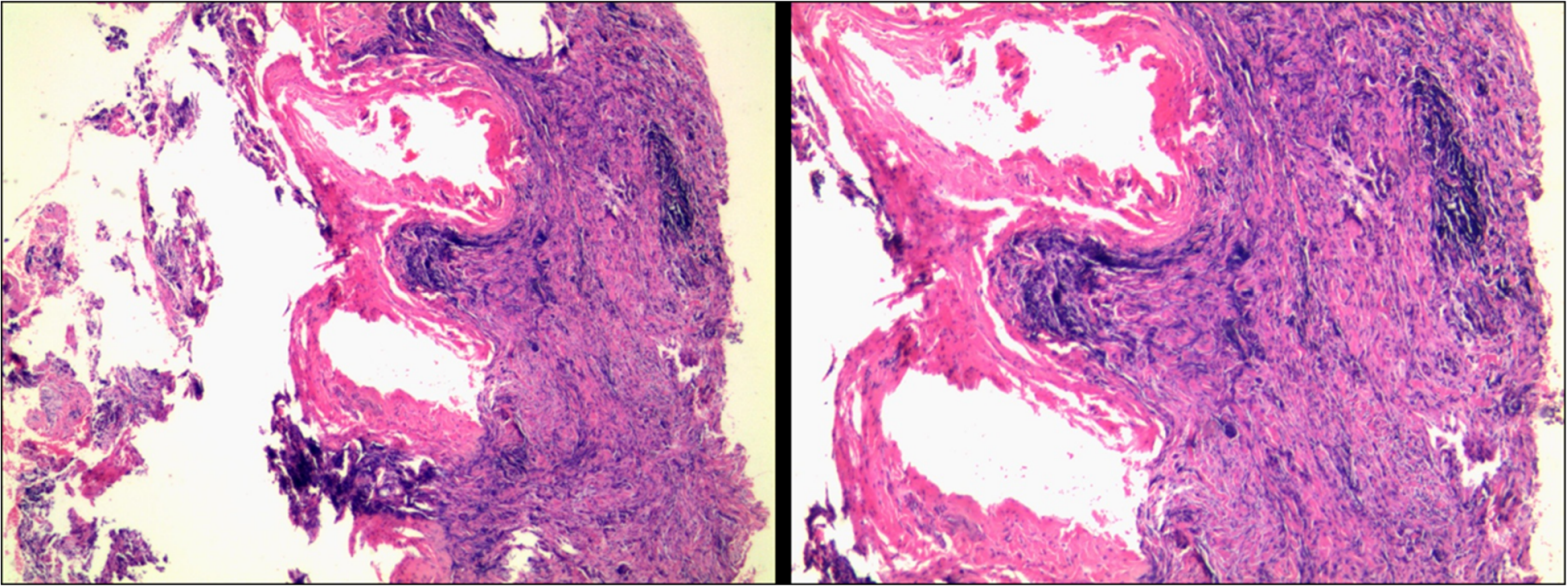
Pathological analysis of the biopsy revealed a hemangioma

Computed tomography (CT) revealed that the bilateral frontal bone, right temporal bone and right parietal bone were diffusely and osteolytically destroyed with soft tissue lesions. (Fig. 1). No metastatic lesions were observed on the CT images of the chest, abdomen, and pelvis. The magnetic resonance imaging (MRI) results revealed that the bilateral frontal bone, right temporal bone, and right parietal bone were thickened with nodules. The lesions were tent-like on coronal and sagittal planes (Fig. 2e, f). The lesions exhibited a slight hypointensity on T1-weighted imaging (T1WI) (Fig. 2a), and isointensity on T1-weighted imaging (T2WI) (Fig. 2b) and T2-fluid-attenuated inversion recovery (FLAIR) (Fig. 2c) images, and were significantly enhanced (Fig. 2d, e, f) after enhancement. The meninges of the right frontal, temporal, and parietal areas were also diffusely thickened with nodules. The nodules compressed the right temporal lobe slightly and led to edema of the parenchyma (Fig. 2a, b, c), which was not enhanced (Fig. 2d, e). There was slight compression of the adjacent brain parenc hyma and ventricle. The brain midline structure shifted to the left by approximately 0.5 cm, and the size of the meningeal nodular lesions was approximately 2.8 × 1.3 × 2.8 cm. The tumor marker levels were: carcinoembryonic antigen (CEA) 11.52 ng/ml, cancer antigen 15–3(CA15–3) 77.39 U/m l. The results combined with the medical history, led us to suspect the presence of a metastatic tumor with invasion of the right temporal and occipital meninges. Systemic bone imaging revealed abnormal bone metabolism in the skull (Fig. 3). To evaluate the nature of the lesion, open biopsy was performed. The pathological diagnosis of the biopsy was hemangioma of the skull (Fig. 4). The corrected diagnosis was cranial cavernous hemangioma.

**Fig. 5:**
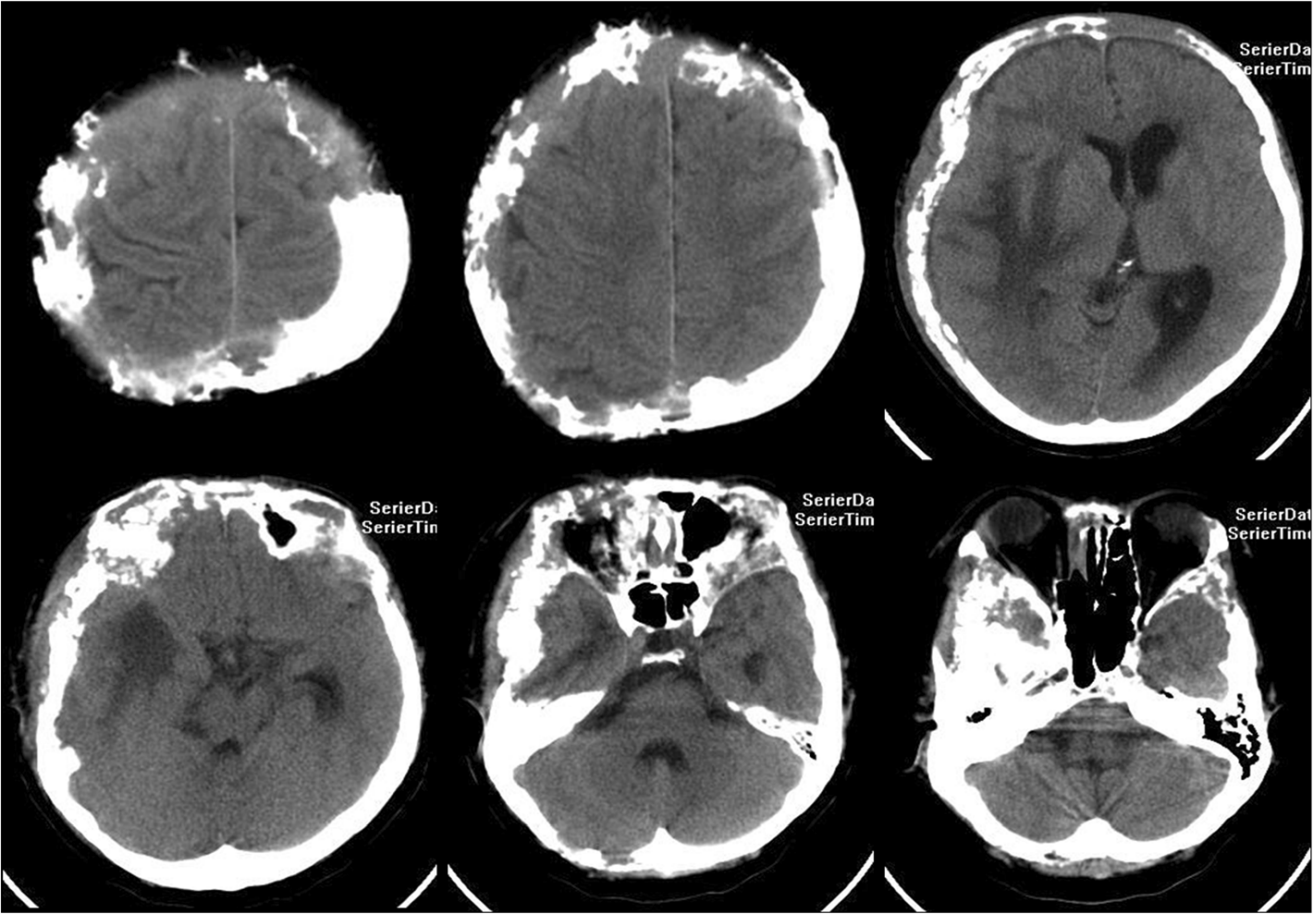
The head computed tomography scan which was performed at the end of radiation therapy revealed no significant change compared to that before radiotherapy

Based on the CT and MRI findings, the headache and upper eyelid edema of the patient were considered to be caused by the local invasion of the skull lesions. The patient received a normal three-dimensional conformal radiotherapy with a dose of 60Gy/30F (the lesions nearby bilateral upper orbital bone was 50Gy/30F). The target volume was described as: according to CT and MRI images to delineate the target, gross tumor volume (GTV) comprised the skull, meningeal and Right temporal lobe lesions, GTV was enlarged 3 mm circumferentialy to form the planning target volume (PTV). Irradiation was performed using 6 MV X-rays. At the end of the radiotherapy, the size of the lump at biopsy site (the primary right frontal, temporal, and top scalp) was slightly reduced on physical examination, but the head CT showed no significantly change compared to pre-radiotherapy CT images (Fig. 5). The symptoms of discomfort in the head and eye swelling were relieved. We concluded that the blood vessels of lesions in skull may be blocked. The patient reported no symptoms during the 1-year telephonic follow-up. Unfortunately, the CT scan during 1-year follow-up was performed at an outside institution and was not available for inclusion in this report.

## Discussion and conclusions

Primary hemangiomas could be divided into three types: cavernous, capillary, and mixed. Cavernous hemangioma is the most common type in the skull, accounting for 10% of all benign tumors and 0.2% of cranial tumors of the skull [3]. Hemangiomas could occur in any part of the skull, such as the clivus [4, 5], frontal bone [6, 7], parietal bone or petrous bone [8], but are predominantly located in the frontal bone, with 44.1% of skull PICHs reported in this area in previous studies [2]. To date, the origin of hemangiomas remains unclear. Some researchers have proposed that cavernous hemangioma may be an inherited disease, and a mutation in KRIT1/CCM1 gene which encodes a protein that affects endothelial cell structure and function is correlated with the pathogenic mechanisms of cavernous hemangioma [9]. Recent studies have reported that trauma and ionizing radiation particularly treatment with stereotactic radiosurgery (SRS) for brain tumors may be related to the development of cavernous hemangioma [10–12]. It was worth mentioning that our patient received radiotherapy of the right chest wall combined regional > 10 years prior due to breast cancer. Hemangiomas of the skull primarily present as a single mass, with a few cases of multiple masses [13, 14]. Hemangiomas of the skull typically occur in patients in the fourth and fifth decades of life, and the incidence rate in men and women is approximately 1:2–3 [15]. Hemangiomas of the skull originate from the plate barrier, and spread along both sides of the barrier, which is indicative of an osteolytic mass [2]. Hemangiomas of the skull are predominantly solitary, and rarely invade the entire skull. To our knowledge, we report the first case of a diffuse cavernous hemangioma of the skull, wherein the CT and MRI results lead to the misdiagnosis of skull metastasis. Hemangiomas of the skull have no typical clinical symptoms: some patients present with chronic headaches, while some have no significant complaints aside from a local swelling [16].

Cavernous hemangiomas consist of clusters of dilated blood vessels, which are separated by fibrous septa, whereas capillary ones are rich in small vascular luminas without a significant number of fibrous septa. Radiological evaluation includes standard skull X-rays, CT scans, and MRI. X-ray is the most basic method of examination, while CT is the most commonly used method of inspection. However, PICHs of the skull do not have typical radiological features. Non-enhanced CT typically reveals a mass, which represents an osteolytic lesion, with intense enhancement after intravenous contrast administration. MRI of the lesion was inconclusive on T1-weighted images with isointensity or hyperintensity, and irregular hyperintensity on T2 weighted images [17]. The lesion exhibited intense homogenous postcontrast enhancement.

For a single mass, complete resection was beneficial, and the cosmetic results of most cases were satisfactory and revealed no recurrence within a short period of time [18, 19]. If the cranial cavernous hemangioma was relatively large, embolization combined with craniotomy may be the superior treatment [20]. However, multiple lesions may require combined radiotherapy, particularly for diffuse cranial hemangiomas. Such as this case, the treatment efficacy of radiotherapy which relieved the discomfort symptoms of the patient was satisfactory, although skull lesions had no significant change in size. Given the condition that long-term radiographic imaging was helpful for assessing the efficacy of radiation therapy for PICHs, the patient was advised to attend regular follow-up assessments. In addition, it is well known that tumors result from the abnormal expression of genes events, and some recent studies have reported that mutation of some genes was related to development of hemangiomas [21, 22]. If we could perform gene detection in cases of hemangioma of the skull, and then recommend targeted therapy, it could be a prominent topic in future studies.

In this case, the patient received surgery for mucinous breast carcinoma in 2004, and received postoperative chemoradiotherapy, which led to the misdiagnosis of metastasis. In addition, hemangiomas of the skull often grow slowly, over several months to several years, the symptoms are not typical, and often present as a single mass or multiple lesions with osteolytic lesions [13, 14]. In this case, although the intraosseous lesion of the skull presented as an osteolytic lesion, the CT and MRI images indicated that the lesion was diffuse. In addition, some enhanced nodules with variable surrounding vasogenic edema were observed on the right side of the temporal and temporal occipital meninges, which presented occupying lesion making the brain midline structure shift to the left. Furthermore, the CT image revealed diffuse osteolytic bone destruction, which seemed to be a “bean curd” type of change, and the border of the lesion was not clear. All of the above characteristics were similar to those of metastatic lesions [23, 24]. Furthermore, the level of the tumor marker, CA15–3, was higher. It is well-known that more patients with metastatic breast cancer have elevated CA15–3 levels. Based on the above, metastasis of the skull was diagnosed. However, the location of the lesions, which were mainly in the skull, was rare in cases of metastatic lesions. Thus, the scalp thickening lesions were biopsied, and the pathological diagnosis was hemangioma of the skull. The lesion of the cranial hemangioma grew rapidly and the local surface irregularity was apparent. After radiotherapy, the size of the lump at biopsy site was reduced and the distending pain of the head was alleviated. CA15–3 may be considered an early warning sign and was found to be indicative of a high risk of recurrence in breast cancer patients. For this case, CA15–3 may be an early warning sign of recurrence of breast cancer. Therefore, the patient was advised to attend regular follow-up assessments.

In conclusion, PICH of the skull are rare benign lesions characterized by osteolytic bone destruction and have no typical clinical symptoms. Diffuse cavernous hemangioma of the skull is exceedingly rare, and imaging findings are not specific, which leads to misdiagnosis. Pathological evaluation is therefore necessary and important. In cases wherein the mass could not be completely removed by surgery, and radiotherapy would be beneficial.

## Abbreviations

CA15–3: Cancer antigen 15–3
CEA: Carcinoembryonic antigen
CMF: Cyclophosphamide+methotrexate+fluorouracil
CT: Computed tomography
FLAIR: Fluid-attenuated inversion recovery
GTV: Gross tumor volume
MRI: Magnetic resonance imaging
PICHs: Primary intraosseous cavernous hemangiomas
PTV: Planning target volume
SRS: Stereotactic radiosurgery
T1WI: T1-weighted imaging
T2WI: T1-weighted imaging
